# Rapid Isolation of Rare Isotype-Switched Hybridoma Variants: Application to the Generation of IgG2a and IgG2b MAb to CD63, a Late Endosome and Exosome Marker

**DOI:** 10.3390/antib9030029

**Published:** 2020-07-02

**Authors:** Stéphanie Charrin, Roberta Palmulli, Martine Billard, Denis Clay, Claude Boucheix, Guillaume Van Niel, Eric Rubinstein

**Affiliations:** 1 Centre d’Immunologie et des Maladies Infectieuses, Inserm, CNRS, Sorbonne Université, CIMI-Paris, 75013 Paris, France; Stephanie.charrin@inserm.fr; 2 Centre National de la Recherche Scientifique, Structure and Membrane Compartments, Institut Curie, Paris Sciences & Lettres Research University, UMR144, 75005 Paris, France; rp681@cam.ac.uk; 3 Modèles de cellules souches malignes et thérapeutiques, Inserm, Université Paris-Saclay, 94800 Villejuif, France; mbillard@hotmail.fr (M.B.); claude.boucheix@inserm.fr (C.B.); 4 Inserm, Université Paris-Saclay, UMS44, F-94800 Villejuif, France; denis.clay@inserm.fr; 5 Institute of Psychiatry and Neuroscience of Paris (IPNP), Inserm, Université de Paris, U1266, F-75014 Paris, France; guillaume.van-niel@inserm.fr

**Keywords:** CD63, exosomes, late endosomes, isotype switch

## Abstract

CD63, a member of the tetraspanin superfamily, is used as a marker of late endosomes and lysosome-related organelles, as well as a marker of exosomes. Here, we selected rare isotype variants of TS63 by sorting hybridoma cells on the basis of their high expression of surface immunoglobulins of the IgG2a and IgG2b subclass. Pure populations of cells secreting IgG2a and IgG2b variants of TS63 (referred to as TS63a and TS63b) were obtained using two rounds of cell sorting and one limited dilution cloning step. We validate that these new TS63 variants are suitable for co-labeling with mAb of the IgG1 subclass directed to other molecules, using anti mouse subclass antibodies, and for the labeling of exosomes through direct binding to protein A-coated gold particles. These mAbs will be useful to study the intracellular localization of various proteins and facilitate electron microscopy analysis of CD63 localization.

## 1. Introduction

CD63 is a member of the tetraspanin superfamily, proteins characterized by four transmembrane domains and a specific fold in the largest of the two extracellular domains [1,2]. CD63 plays a role in protein and membrane trafficking. On the one hand, it regulates the trafficking of other proteins such as the β-chain of the gastric H+/K+-ATPase, CXCR4, to intracellular compartments [3,4]. On the other hand, it regulates membrane trafficking, for example, the production of intra luminal vesicles in MVB, or IgE-mediated mast cell degranulation [5,6]. CD63 also regulates HIV and papilloma virus infection [7,8]. Mice lacking CD63 have normal lysosomal functions but show a pigmentation defect due to alteration of melanosome biogenesis and a reduction of acute allergic reactions [6,9,10].

CD63 is a ubiquitously expressed protein that was discovered as a protein present on the cell surface of activated blood platelets, known as platelet glycoprotein 40 (Pltgp40). Its expression at the cell surface was later shown to be induced or strongly increased after activation of neutrophils, eosinophils and basophils, following mobilization of the intracellular pool. Its expression is therefore routinely used as an activation and/or degranulation marker of these cells (reviewed in [2,11]).

CD63 is particularly enriched in late compartments of the endosomal pathway, such as multivesicular bodies and lysosomes, and also in other lysosome-related organelles such as azurophil granules of neutrophil granulocytes, dense and α-granules in platelets, melanosomes in melanocytes, cytotoxic granules in T-cells, Weibel–Palade bodies in endothelial cells and major histocompatibility complex II (MHCII) compartments in dendritic cells (reviewed in [2,11]). In multivesicular bodies, CD63 is sorted to intraluminal vesicles that can be released as exosomes after fusion of the multivesicular bodies with the plasma membrane [12,13]. These exosomes represent a new way of intercellular communication that is increasingly involved in most of the patho-physiological pathways and which raise great hope for their use as biomarkers and drug delivery systems [12,13]. Hence, given the wide use of CD63 as a marker of late endosomal compartments and exosomes, new anti-CD63 antibodies with distinct isotypes would provide new opportunities for a variety of technical approaches.

One particular approach that would benefit from the availability of CD63 mAbs of different subclasses is the simultaneous detection of CD63 and other molecules in a single sample. Indeed, multiple labeling can be achieved by using mouse mAbs of different isotypes and secondary polyclonal antibodies directed to these different isotypes, an approach combining both versatility and high sensitivity [14]. However, a major limitation of this approach is the availability of mAbs of different subclasses, notably because the majority of commercially available mouse mAbs are of the IgG1 subclass, reflecting at least in part the representation of this subclass in the circulating serum IgGs in immunized Balb/c mice, the strain most commonly used for generating mouse hybridomas [14]. In this regard, most commercially available CD63 mAbs are of the IgG1 subclass, and to our knowledge none of the few commercially available CD63 mAb of a different isotype has been validated for the specific labeling of late endosomes. There is therefore a need for CD63 mAbs of other subclasses. More generally, the availability of mouse mAbs of different subclasses to markers of various organelles would facilitate the analysis of the distribution of different proteins.

An alternative strategy to the generation of new antibodies is to switch the subclass of existing hybridomas to a different subclass. This can be achieved through recombinant methodologies. Alternatively, one can take advantage of spontaneous isotype class switching of hybridomas. Class switch recombination refers to the change of immunoglobulin subclass that occurs during B cell activation, from IgM to IgG (including IgG1, IgG2a and IgG2b in the mouse), IgE or IgA, allowing antibodies to retain the same antigen specificity but to carry out different effector functions. In this process, intrachromosomal recombination allows the juxtaposition of the rearranged heavy chain V(D)J region that was expressed with the µ constant region to one or another of the downstream constant regions [15]. Myelomas and hybridomas have conserved an ability to switch from one isotype to another in culture making it possible to select for variants that produce a mAb with the same specificity but of different isotypes. However, isotype switching occurs at low frequencies of 10^−5^–10^−7^ [16,17,18], making time-consuming the selection of cells having undergone isotype switching by limited dilution.

We hypothesized that an alternate approach could be to take advantage of the fact that the cell surface immunoglobulin expression correlates with the immunoglobulin secretion rate [19]. Thus it should be possible to select the cells having undergone isotype switching by selecting the cells having the highest surface expression levels of the desired Ig subclass. The feasibility of this approach has been demonstrated for myeloma cells and hybridomas [17,20] but has not been used to our knowledge to change the subclass of antibodies to markers of intracellular compartments. Here, we use this approach to obtain IgG2a and IgG2b variants (respectively TS63a and TS63b) of TS63, an anti CD63 mAb of the IgG1 subclass that was previously generated in our laboratory [21]. We demonstrate the usefulness of these variants for analyzing the colocalization with other molecules recognized by IgG1 mAb, and for the direct labeling of extracellular vesicles using gold beads coated with protein A.

## 2. Materials and Methods

### 2.1. Antibodies

The mAb TS63 directed to CD63 has been previously described [9,21]. It was produced after immunizing BALB/c mice with a mixture of HEL and Jurkat cells. Spleen cells were fused with P3 × 63AG8 mouse myeloma cells according to standard techniques, distributed into 96-well tissue culture plates, and screened by indirect immunofluorescence and immunoprecipitation. Other antibodies were an anti-GM130 (clone 35, BD transduction, IgG1), an anti-EEA1 (clone 14, BD transduction, IgG1), an anti-PDI (RL90, IgG2a, Abcam), an anti-LAMP2 (H4B4, Santa Cruz, IgG1) and an anti-CD9 (TS9, IgG1, produced in our laboratory). The goat anti mouse IgG1, IgG2a and IgG2b antibodies, coupled to Alexa Fluor 488, Alexa Fluor 568 or Alexa Fluor 647 dyes, the goat anti-rabbit Ig coupled to Dylight 650 and the goat anti-mouse Ig coupled to Alexa Fluor 680 were obtained from Thermo Fisher Scientific.

### 2.2. RNA Interference, Western-Blot and Immunoprecipitation.

HeLa cells were plated at the concentration of 25,000/cm^2^ and lysed two days later in RIPA buffer supplemented with protease inhibitors. After 30 min incubation at 4 °C, the insoluble material was removed by centrifugation at 10,000× *g*. A 3× concentrated Laemmli buffer was added to a fraction of the lysate for Western-blot, whereas the remaining was incubating with the different antibodies and protein G sepharose beads (GE Healthcare) for 2 h to immunoprecipitate the target antigen. The proteins were separated by SDS-polyacrylamide gel electrophoresis and transferred to a PVDF membrane (GE Healthcare). Western-blotting on lysates was performed using appropriate combinations of primary and Alexa Fluor 680-labeled secondary antibodies. Western-blotting on immunoprecipitations was performed using biotin-labelled antibodies and Alexa Fluor 680-labeled streptavidin. All acquisitions were performed using the Odyssey infrared imaging system (LI-COR Biosciences). RNA interference was performed at the time of plating, using INTERFERin (Polyplus transfection), according to the manufacturer’s protocol. The sequence targeted by the CD63 siRNA is AAGUUCUUGCUCTACGUCCUC. Control siRNA is UUUGUAAUCGUCGAUACCC.

### 2.3. Flow-Cytometry Analysis and Fluorescence-Activated Cell Sorting

HeLa cells were detached, washed twice in complete DMEM and incubated for 30 min at 4 °C with the hybridoma supernatants. After 3 washings, the cells were incubated for 30 min at 4 °C with an Alexa 647 conjugated goat anti mouse antibody. After washing the cells were analyzed using an Accuri C6 flow-cytometer (Becton-Dickinson). For fluorescence activated cell sorting of hybridomas, the cells were incubated with Alexa Fluor 647-coupled goat anti-mouse IgG2a or IgG2b antibodies, or as a control a goat anti-rabbit Ig coupled to Dylight 650 which has spectral properties similar to that of Alexa Fluor 647. Selected cells were sorted using a FacsAria Cell sorter (Beckton Dickinson).

### 2.4. Immunofluorescence and Confocal Microscopy

The cells grown in complete medium were fixed for 15 min with 4% paraformaldehyde at room temperature, washed in PBS and then incubated for 15 min in 50 mM NH_4_Cl in PBS. In most cases, the cells were permeabilized with 0.1% Triton X-100 in PBS for 2 min at 4 °C, and incubated with primary antibodies in PBS supplemented with 0.1% BSA. The binding of primary antibodies was revealed using Alexa Fluor 488 or 568-labelled goat anti mouse Ig subclasses. To preserve the surface labelling of CD9, the Triton X-100 permeabilization step was omitted and the primary and secondary antibody solutions were supplemented with 0.1% saponin. The cells were mounted in Prolong Gold (Thermofisher Scientific) supplemented with DAPI and examined with a Leica SP5 confocal microscope (63× objective, 1.4 numerical aperture, zoom 3 or 8).

### 2.5. Exosome Isolation

Culture media were pre-cleared for extracellular vesicles present in the serum by ultracentrifugation of the culture media at 100,000× *g* ON using a 45Ti rotor and filtered on 0.22 µm filters. Subconfluent MNT-1 cells were grown in pre-cleared media, and culture supernatant was recovered after 48 h. The culture supernatants were sequentially centrifuged at 4 °C at 300× *g* (10 min), 2000× *g* (20 min) and 10,000× *g* for (30 min). Exosomes were collected from the last supernatant by centrifugation at 100,000× *g* for 60 min at 4 °C (rotor 45Ti, 30,000 rpm). The pellet was resuspended and washed in PBS, pH 7.5, as previously described [22].

The pellet was resuspended in PBS (pH 7.5) and mixed with a 40% iodixanol solution, made by mixing a homogenization buffer (0.25 M sucrose, 1 mM EDTA, 10 mM Tris-HCL, (pH 7.4)) and an iodixanol working solution, prepared by combining a working solution buffer (0.25 M sucrose, 6 mM EDTA, 60 mM Tris-HCl, (pH 7.4)) and a stock solution of OptiPrep™ (60% (w/v) aqueous iodixanol solution, Sigma-Aldrich). The gradient was formed by layering 40% (containing exosomes), 20%, 10% and 5% solutions on top of each other in an open top polypropylene tube and centrifuged at 100,000× *g* for 14 h at 4 °C (rotor SW41, Beckman Coulter). Exosome containing fractions (corresponding to a density of 1.08–1.11 g/mL) were collected from the gradient, diluted in PBS and centrifuged at 100,000× *g* for 60 min at 4 °C. The pellet was resuspended in PBS and used for immunogold labeling.

### 2.6. Immunogold Labeling

For immunogold labeling, MNT-1 derived extracellular vesicles were deposited on carbonated EM-grids for 20 min. Then grids were fixed with 2% PFA and processed for single immunogold labelling as reported [23] using TS63a or TS63b and 10 nm protein A gold particles (PAG). The same samples were double labelled using TS63a or TS63b recognized by PAG 10 nm and followed by a labelling using an IgG1 CD9 antibody recognized by 5 nm PAG through a rabbit anti mouse secondary antibody. All samples were analyzed with a Tecnai Spirit electron microscope (FEI Company, Eindhoven Netherlands), and digital acquisitions were made with a numeric camera (Quemesa, EMSIS GmbH, Münster, Germany).

## 3. Results and Discussion

**Figure 1:**
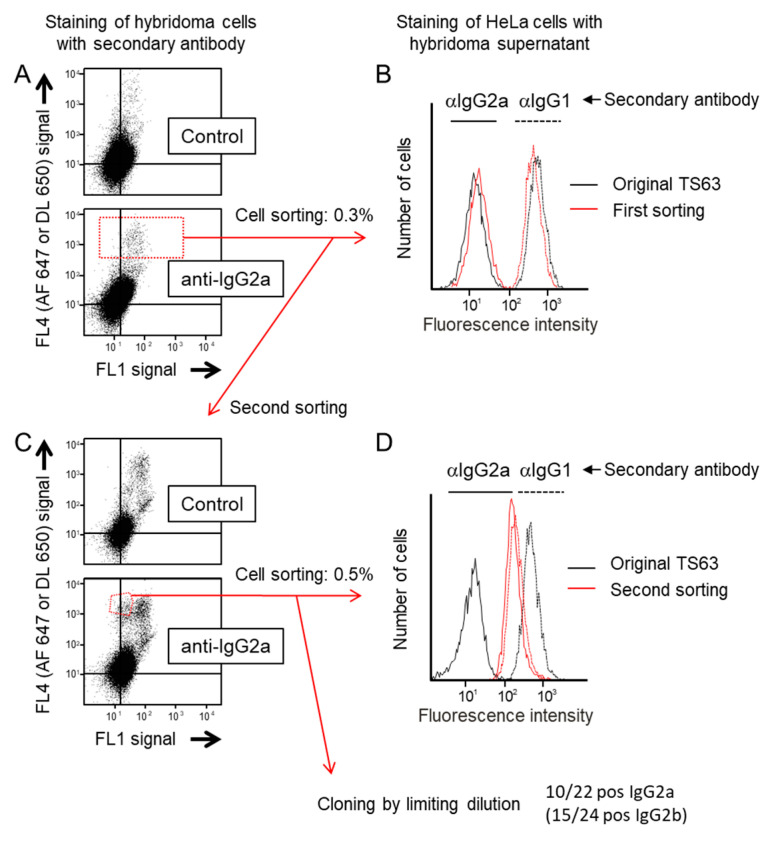
Selection of IgG2a variants of TS63. (**A**) Hybridoma cells were labelled with either a control secondary antibody (a goat anti-rabbit coupled to Dylight 650 (DL 650)) or a goat anti-mouse IgG2a coupled to Alexa Fluor 647 (AF 647). The dot plots show for each cell the value of the Dylight 650 or Alexa 647 fluorescence in the far red Channel (FL4) and that of the autofluorescence in the green channel (FL1). Note that there is no difference between the two labelings indicating that most of the “positive” signal is non-specific. The gate used to sort the cells with the highest level of staining is drawn in the bottom dot-blot. (**B**) After being grown for a few days, the supernatant of the sorted cells was used to stain HeLa cells by indirect immunofluorescence, using either anti (α)-IgG1 or anti-IgG2a polyclonal antibodies coupled to Alexa 647 as secondary reagents. A staining with the conditioned medium of parental TS63 cells was performed in parallel. The fluorescence staining of the cells was analyzed by flow-cytometry. Note that the supernatant of sorted cells stains HeLa cells slightly better than that of parental cells when the binding of the mAb is revealed by an anti-IgG2a antibody. (**C)** After amplification, the cells sorted in A were subjected to a second sorting. Note that there is a specific cell population uniquely detected by the anti-mouse IgG2a labeling. The gate used to sort the cells is drawn in the bottom dot-blot. (**D**) Labelling of HeLa cells as in B after growing the cells sorted the second time for a few days. Note that the staining of HeLa cells by the supernatant of the sorted cells is similar whether the binding of the mAb is revealed by an anti-IgG2a antibody or an anti-IgG1 antibody.

To select cells expressing IgG2a or IgG2b at their cell surface, the hybridoma cells producing the TS63 mAb were incubated with Alexa 647-labelled goat anti-mouse IgG2a or IgG2b, or a Dylight 650-labelled goat anti-rabbit antibody as a control, and the intensity of labelling was analyzed by flow-cytometry. As exemplified for the staining of IgG2a in Figure 1A, there was no major difference between the labelling with the anti-subclass antibodies and the control antibody, indicating that most of the “positive” signal observed was non-specific. The cells displaying the highest level of staining (~0.3%) with the anti-IgG2a or anti-IgG2b mAbs were sorted and grown until sufficiently concentrated for further analysis.

The enrichment of TS63 variants of the IgG2a and IgG2b subclasses was tested by comparing by flow-cytometry the staining intensity of HeLa cells with the conditioned medium of sorted TS63 cells with that of parental TS63 cells, using anti IgG2a, IgG2b or IgG1 secondary antibodies. As shown in Figure 1B, there was a slight increase in the labelling of HeLa cells by the supernatant of anti IgG2a-sorted TS63 cells, using the anti-IgG2a antibodies. This was not due to a lower mAb concentration in the parental TS63 medium, because this medium gave a higher staining when the secondary reagent was an anti-IgG1 antibody. Thus, this first sorting slightly enriched the culture in IgG2a-secreting cells. Similar results were obtained for the selection of IgG2b-secreting cells (not shown).

To further enrich the culture in cells secreting the desired subclass, a second cell sorting was performed after labelling the hybridoma cells under the same condition. As shown in Figure 1C, a new population of TS63 cells was present after labelling with the anti-IgG2a Ab but not after labelling with the control Ab. This population strongly labelled with the anti-IgG2a antibody had a lower green auto-fluorescence intensity (FL1) than the other strongly labelled cells (which were also present in the control sample), suggesting that these other cells may correspond to damaged cells. We isolated this new cell population (~0.5%) and after a few days of culture tested their supernatant for the labelling of HeLa cells. As shown in Figure 1D, the conditioned medium of these cells clearly stained HeLa cells even when an anti IgG2a Ab was used as a secondary reagent, indicating a strong enrichment of cells producing an IgG2a variant of TS63. Similarly, a second sorting using anti-IgG2b Ab enriched the culture in IgG2b-producing cells (data not shown). In order to obtain a pure population of cells secreting the IgG2a or IgG2b variants, the cells obtained after the second sorting were cloned by limited dilution and selected based on the binding to HeLa cells and recognition by either anti-IgG2a or IgG2b antibodies. Ten out of 22 clones tested were IgG2a, and 15 out of 24 were IgG2b. Considering the fraction of cells selected in the two sortings, the percent of switch variants in the original cell culture was determined to be between 10^−5^ and 10^−6^, consistent with previous reports [16,17,18].

**Figure 2:**
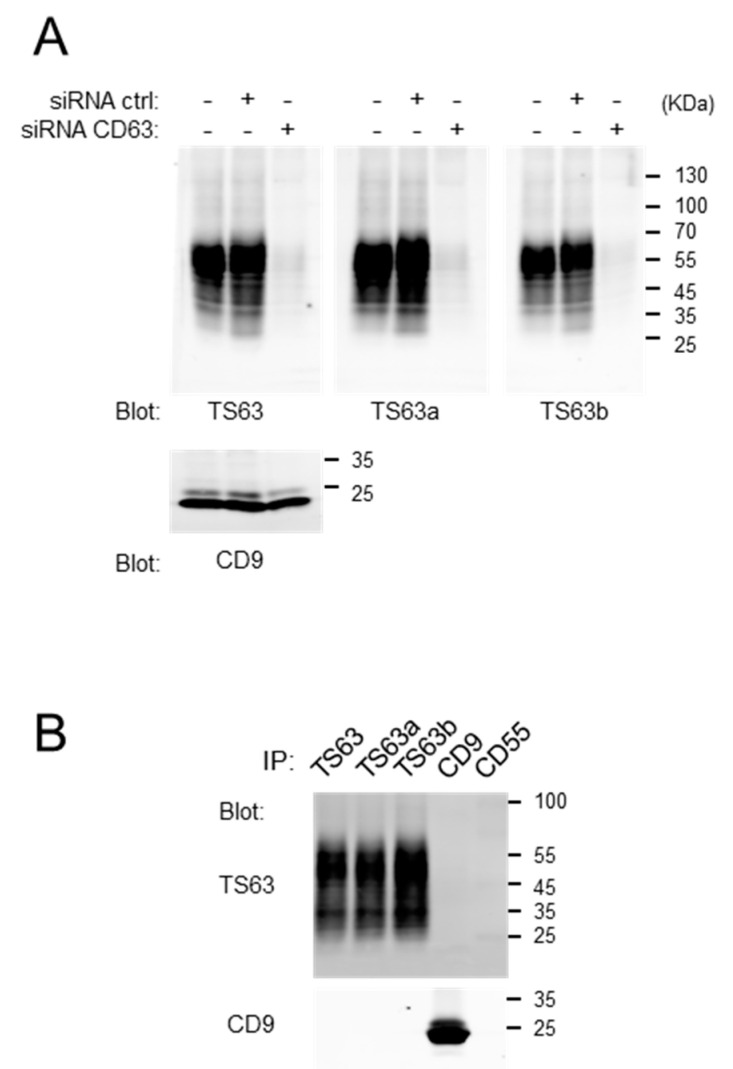
Validation that the TS63 variants recognize CD63. (**A**) Western-blot analysis, using the different TS63 variant or a CD9 mAb as a control, of HeLa cell lysates after treatment or not with a siRNA targeting CD63 or a control siRNA. (**B**) Immunoprecipitations using the different TS63 variants or CD9 and CD55 mAb as a control. The presence of CD63 and CD9 in the immunoprecipitates was analyzed by Western-blot using biotin-labeled TS63 and TS9 mAbs.

One clone of each subclass was selected for further analysis. We verified that the new variants, TS63a (IgG2a) and TS63b (IgG2b), kept the properties of the parental TS63 mAb. As shown in Figure 2, both antibodies strongly recognized CD63 by Western-blot in control cells, but not in cells depleted of CD63 by RNA interference and were able to immunoprecipitate CD63 with a similar efficiency to that of TS63.

**Figure 3:**
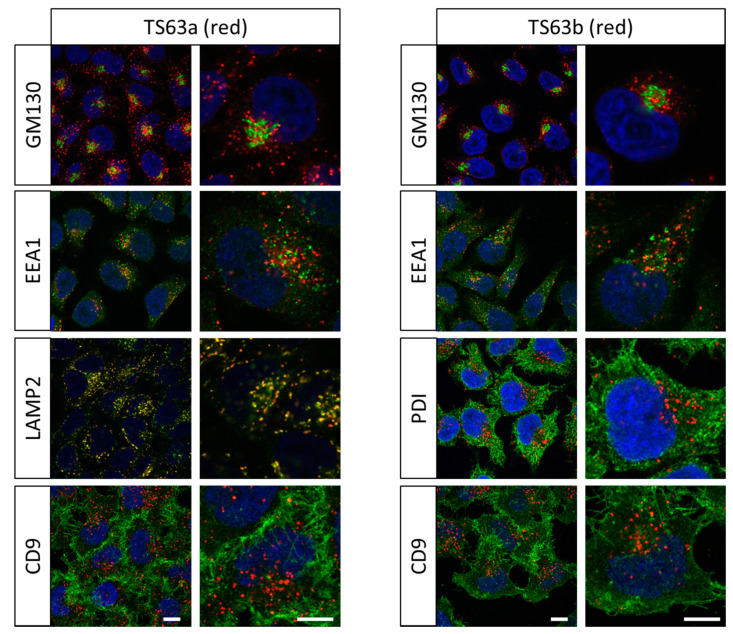
Confocal microscopy analysis of CD63 distribution and comparison with other organelle markers. HeLa cells were grown for two days on coverslips, fixed, permeabilized and stained with a combination of either TS63a or TS63b, and mAbs of different subclasses to markers of different compartments. The binding of the antibodies was revealed using goat polyclonal antibodies to mouse Ig subclasses coupled to AlexaFluor 488 or AlexaFluor 568. GM130: cis-Golgi; EEA1, early endosome; LAMP2: late endosome; PDI: endoplasmic reticulum; CD9: plasma membrane. Bar: 10 µm.

We then tested whether these antibodies can be used as organelle markers in combination with IgG1 antibodies to other target molecules, and using anti IgG2a, IgG2b and IgG1 secondary antibodies. For this purpose, we labeled permeabilized HeLa cells with TS63a or TS63b mAbs and selected mAbs to various compartments. As shown in Figure 3, the labelling of CD63 with either TS63a or TS63b gave a different staining pattern from that obtained with IgG1 mAbs to markers of the cis-Golgi (GM130), early-endosome (EEA1) or the plasma membrane (CD9). The staining of TS63b was also different from the staining of a marker of the endoplasmic reticulum (PDI) recognized by a mAb of the IgG2a subclass. In contrast, and as expected, the staining with TS63a strongly overlapped with that of another late endosome marker (LAMP2, labelled with an IgG1 mAb). Thus, TS63a and TS63b can be used to compare the distribution of proteins recognized by mouse IgG1 mAbs with the localization of CD63-positive late endosomes.

**Figure 4:**
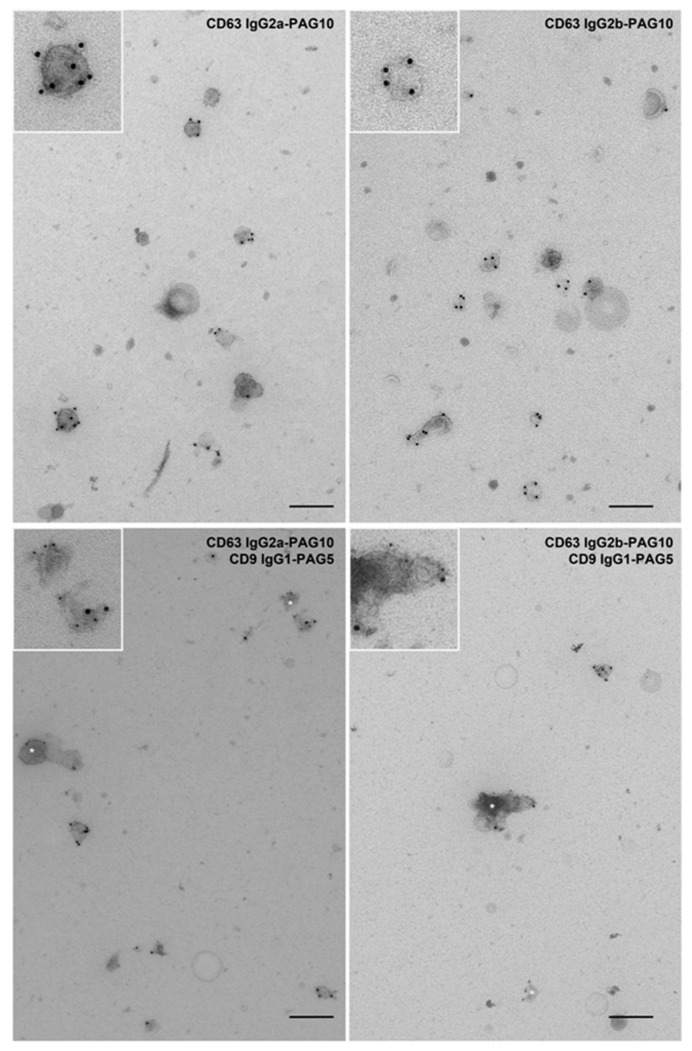
Immunogold labelling of extracellular vesicles with TS63a and TS63b.Top panels: TS63a or TS63b were used to label extracellular vesicles derived from MNT-1 cells. Both antibodies revealed specific and efficient labelling of a proportion of extracellular vesicles. Bottom panels: TS63a or TS63b were used to co-label extracellular vesicles derived from MNT-1 cells with anti-CD9 antibody. Both antibodies displayed distinct and specific labelling for each tetraspanin (10 nm gold particles for CD63 and 5 nm particles for CD9) and revealed subpopulations of extracellular singly labelled for either CD63 or CD9 (indicated by white star) or co-labelled for both. Inset shows a zoom-in of each panel. Scale bar: 200 nm.

We finally tested whether TS63a and TS63b can be used for single and double immunogold labeling of extracellular vesicles for electron microscopy observation. IgG2a and IgG2b isotypes have the benefit to efficiently bind to protein A coupled to gold particles in contrary to IgG1 primary antibodies that need to be linked to protein A via a “bridge” antibody such as a rabbit anti-mouse antibody, hence reducing both the risk of background staining and the time required to perform the staining. We first labeled extracellular vesicles isolated from the culture supernatant of the human melanocytic cell line MNT-1, which are enriched in CD63 [24]. As shown in Figure 4, both isotype revealed a specific labeling of a subpopulation of vesicles with a high efficiency. Pellets of isolated extracellular vesicles often consist of subpopulations of vesicles that can display subtle differences in terms of composition [25,26]. It is therefore of interest to co-label extracellular vesicles to distinguish sub-populations. We then tested whether TS63a and TS63b can be used in combination with IgG1 antibodies targeting CD9, a tetraspanin also enriched in extracellular vesicles. Double immunogold labeling of MNT-1 derived extracellular vesicles showed a specific labeling of each isotype and revealed subpopulations only labeled for CD63 or CD9 and subpopulations co-labeled by antibodies against CD63 and CD9. Thus, TS63a and TS63b can be used to label extracellular vesicles and to compare the distribution CD63 on these vesicles with that of proteins recognized by mouse IgG1 mAbs.

## 4. Conclusions

We were able to generate in a few weeks and with limited manipulations IgG2a and IgG2b variant of TS63, a mAb directed to CD63, a marker of late endosomes, lysosome-related organelles as well as exosomes. We have also demonstrated the interest of these new mAbs for the analysis of the distribution of proteins recognized by mAbs of the IgG1 subclass, whether by confocal microscopy or electron microscopy. These mAbs may be useful for a variety of additional technical approaches such as ELISA or flow cytometry analysis. They may be used for example to analyze by multi-color flow cytometry the activation state of mast cells or other pertinent cells together with the level of expression of other proteins when only unlabeled IgG1 mAbs to these proteins are available. Applying the approach used in this study to generate antibodies of different subclass to other mAbs directed to various organelle markers may considerably increase the possibility of performing multi-color labelling using anti mouse IgG isotypes as secondary reagents.
